# Vaginal microbiota are associated with in vitro fertilization during female infertility

**DOI:** 10.1002/imt2.185

**Published:** 2024-03-19

**Authors:** Tao Wang, Penghao Li, Xue Bai, Shilin Tian, Maosen Yang, Dong Leng, Hua Kui, Sujuan Zhang, Xiaomiao Yan, Qu Zheng, Pulin Luo, Changming He, Yan Jia, Zhoulin Wu, Huimin Qiu, Jing Li, Feng Wan, Muhammad A. Ali, Rurong Mao, Yong‐Xin Liu, Diyan Li

**Affiliations:** ^1^ Antibiotics Research and Re‐evaluation Key Laboratory of Sichuan Province, Sichuan Industrial Institute of Antibiotics, School of Pharmacy Chengdu University Chengdu China; ^2^ Jinxin Research Institute for Reproductive Medicine and Genetics, Sichuan Jinxin Xi'nan Women's and Children's Hospital Chengdu China; ^3^ Shenzhen Branch, Guangdong Laboratory of Lingnan Modern Agriculture, Genome Analysis Laboratory of the Ministry of Agriculture and Rural Affairs, Agricultural Genomics Institute at Shenzhen Chinese Academy of Agricultural Sciences Shenzhen China; ^4^ College of Animal Science and Technology Sichuan Agricultural University Chengdu China; ^5^ College of Life Sciences Wuhan University Wuhan China; ^6^ College of Food and Biological Engineering Chengdu University Chengdu China; ^7^ College of Agriculture Kunming University Kunming China; ^8^ State Key Laboratory of Southwestern Chinese Medicine Resources Chengdu University of Traditional Chinese Medicine Chengdu China; ^9^ School of Biological Sciences University of the Punjab Lahore Pakistan

**Keywords:** community, infertility, IVF, vaginal microbiome

## Abstract

The vaginal microbiome plays an essential role in the reproductive health of human females. As infertility increases worldwide, understanding the roles that the vaginal microbiome may have in infertility and in vitro fertilization (IVF) treatment outcomes is critical. To determine the vaginal microbiome composition of 1411 individuals (1255 undergoing embryo transplantation) and their associations with reproductive outcomes, clinical and biochemical features are measured, and vaginal samples are 16S rRNA sequenced. Our results suggest that both too high and too low abundance of *Lactobacillus* is not beneficial for pregnancy; a moderate abundance is more beneficial. A moderate abundance of *Lactobacillus crispatus* and *Lactobacillus iners* (~80%) (with a pregnancy rate of I‐B: 54.35% and III‐B: 57.73%) is found beneficial for pregnancy outcomes compared with a higher abundance (>90%) of *Lactobacillus* (I‐A: 44.81% and III‐A: 51.06%, respectively). The community state type (CST) IV‐B (contains a high to moderate relative abundance of *Gardnerella vaginalis*) shows a similar pregnant ratio (48.09%) with I‐A and III‐A, and the pregnant women in this CST have a higher abundance of *Lactobacillus* species. Metagenome analysis of 71 samples shows that nonpregnant women are detected with more antibiotic‐resistance genes, and Proteobacteria and Firmicutes are the main hosts. The inherent differences within and between women in different infertility groups suggest that vaginal microbes might be used to detect infertility and potentially improve IVF outcomes.

## Highlights

- Pregnancy rates vary among women with different vaginal microbiome communities.
- Too high abundances of both *Lactobacillus crispatus* and *Lactobacillus* *iners* have negative effects on in vitro fertilization (IVF) outcomes.
- A moderate abundance (around 80%) of *Lactobacillus* is more beneficial for pregnancy.

## Graphical abstract

The vaginal microbiome is crucial for reproductive health, and as global infertility rates rise, understanding its impact on in vitro fertilization (IVF) is vital. Here, we analyzed the microbiome of 1411 individuals (1255 undergoing embryo transplantation) and revealed that a moderate abundance of *Lactobacillus* (~80%) is most beneficial for pregnancy (pregnancy rates were 54.35% and 57.73% for community state types [CST] I‐B and III‐B respectively), outperforming higher abundances (>90%) seen in I‐A (44.81%) and III‐A (51.06%). Metagenomic analysis of 71 samples indicates more antibiotic‐resistance genes in nonpregnant women, with Proteobacteria and Firmicutes as predominant hosts. Variations among infertility groups suggest potential applications for detecting infertility and enhancing IVF outcomes using vaginal microbes. AMH, antimullerian hormones; BMI, body mass index; E2, estradiol; FSH, follicle‐stimulating hormone; LH, luteinizing hormone; P, progesterone; PRL, prolactin; T, testosterone.

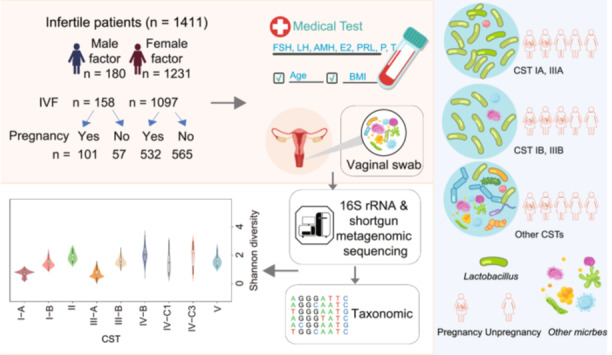

## INTRODUCTION

Female infertility presents itself as a multifaceted health concern, stemming from single or multiple factors that hinder reproductive capabilities. It affects around 2%–0.5% women between the ages of 20 and 44, leading to primary and secondary infertility. This condition may result from a blend of congenital and hormonal irregularities, lifestyle choices, and environmental factors [1]. The well‐known causes of female infertility are ovulation problems, polycystic ovary syndrome, fallopian tube problems, uterus problems, and endometriosis [2, 3]. These causes are also believed to be associated with the imbalance of reproductive microbiota [4]; an altered reproductive tract microbiota may contribute to female infertility [5]. The prevalence of infertility is rising [6], and assisted reproductive technologies (ART) are increasingly in demand, as well as becoming safer and more successful [7]. In vitro fertilization (IVF) procedures, aimed at overcoming infertility and achieving a successful pregnancy, have been globally employed for nearly four decades.

The vaginal microbiota plays an important role in maintaining vaginal health and protecting the host from diseases [8, 9]. Vaginal microbiota can be affected by host physiology and can also affect the host reproductive physiology itself [8, 10]. There is increasing interest in identifying the microbiota that specifically impacts female reproductive health and the health of offspring. Recognized vaginal pathogens such as *Mycoplasma tuberculosis*, *Chlamydia trachomatis*, and *Neisseria gonorrhoeae* have the potential to lead to infertility [11]; subclinical changes in the microbiota leading to bacterial vaginosis (often caused by *Gardnerella vaginalis*) are also thought to be associated with an increased risk of miscarriage [12]. These studies indicate a growing likelihood that infertile patients possess distinct reproductive tract microbiota (lower and/or upper) when compared to healthy and fertile women [13]. In addition to vaginal health, the composition of the vaginal microbiome also appears to be linked to the likelihood of natural conception and ART cycle success [14].

**Figure 1:**
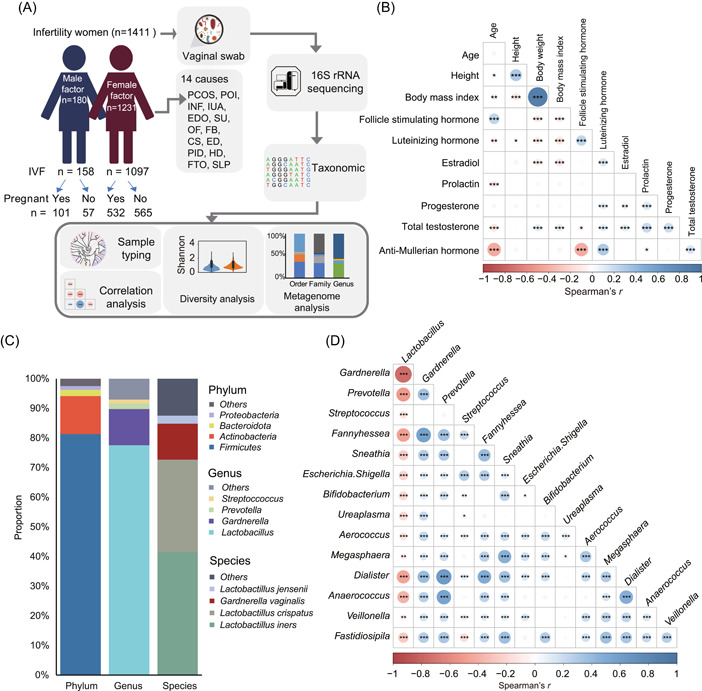
Correlations among different measurements and composition of the vaginal microbiota. (A) Overview of the workflow for integrated analysis of the sample information, vaginal microbiota, and phenotype in 1411 infertile women. (B) Spearman correlations among 11 measurements. The size of the circle represents |*r*|, and the statistical significance of the *p* values was calculated using two‐sided hypothesis testing. (C) Composition of the vaginal microbiota at different levels (namely phylum, genus, and species) according to the median relative abundance. Taxa that made up <1% of the microbiota were combined and labeled as “Others.” (D) Spearman correlations among different genera. *p < 0.05, **p < 0.01, and ***p < 0.001. CS, chronic salpingitis; ED, endometritis; EDO, endometriosis; FB, fallopian problem; FTO, fallopian tube obstruction; HD, hydrosalpinx; INF, inflammatory; IUA, intrauterine adhesion; IVF, in vitro fertilization; OF, ovulation failure; PCOS, polycystic ovarian syndrome; PID, pelvic inflammatory disease; POI, premature ovarian insufficiency; SLP, salpingectomy; SU, scarred uterus.

Thus, it is important to consider whether IVF outcomes could be influenced by the microbial composition of the reproductive tract among women with different types of infertility. Research has shown that the vaginal microbiome may predict the outcome of IVF [15]. In this study, we systematically sampled the vaginal microbiota of 1411 females with infertility from a large cohort of women of reproductive age (Figure 1A and Table S1). Bacterial communities were profiled with 16S rRNA gene amplicon sequencing (Table S2), and 71 females were further metagenome sequenced (Table S3). The analysis revealed correlations between clinical and biochemical parameters and infertility, identifying microbiome signatures associated with these clinical and biochemical measurements. Potential bacterial markers for good IVF outcomes were also identified.

## RESULTS

### Phenotype and vaginal microbial composition

The biochemical and clinical factors of 1411 females diagnosed with infertility for no pregnancy, the age averaged 31.66 (±5.21 standard deviation, SD) and body mass index (BMI) averaged 22.33 (±3.28 SD), were analyzed (Table S1). Age and levels of luteinizing hormone (LH), prolactin (PRL), and antimullerian hormones (AMH) were negatively correlated (*p* < 0.05) but positively correlated with follicle‐stimulating hormone (FSH) after multiple testing corrections. Serum PRL levels were also positively correlated with AMH (Figure 1B). From 1411 vaginal samples, the 16S rRNA gene sequencing analysis produced a total of 112,635,137 high‐quality sequences, with an average of 79,826 reads per sample (Table S2), and the samples had an average good coverage of 99.83%. A total of 1550 amplicon sequence variants (ASVs) corresponding to the operational taxonomic unit (OTU) representative sequence were identified with a 100% similarity clustering (Table S4). We subsequently classified the ASVs into 446 genera, 215 families, 137 orders, 68 classes, and 35 phyla. After removing contaminants and retaining taxa that exhibited an abundance of at least 1% in at least 15% of samples, 15 genera and 26 species were used for further analysis. In women of reproductive age, Firmicutes were the most prevalent at the phylum level, followed by Actinobacteria and Bacteroidetes. At the genus level, the vaginal flora of reproductive‐age women was primarily composed of *Lactobacillus*, followed by *Gardnerella*, *Prevotella*, *Streptococcus*, and *Fannyhesea*. Various *Lactobacillus* species (~77.57% of relative abundance) were the dominant bacteria in the vagina of reproductive‐age women. At the species level, we detected the following species belonging to the *Lactobacillus* genus: *Lactobacillus iners* (41.54%), *Lactobacillus crispatus* (31.12%), *Lactobacillus jensenii* (2.63%), and *Lactobacillus* *hominis* (1.41%). For the genus *Gardnerella*, the most abundant species of this genus is *G. vaginalis* (12.21%) (Figure 1C). For the 15 genera and 26 species present in at least 15% of the individuals with an abundance greater than 0.8%, we found a significantly widespread presence of *Lactobacillus*, which was all negatively correlated with the abundance of other 14 genera, respectively (*p* < 0.05). *Gardnerella* and *Prevotella* were significantly positively correlated with 13 and 11 genera, respectively (*p* < 0.05) (Figure 1D). At the species level, *L. iners* and *L. crispatus* were found to be negatively correlated with the abundance of most other species (Figure S1).

### Community types of vaginal microbiota in infertility women

**Figure 2:**
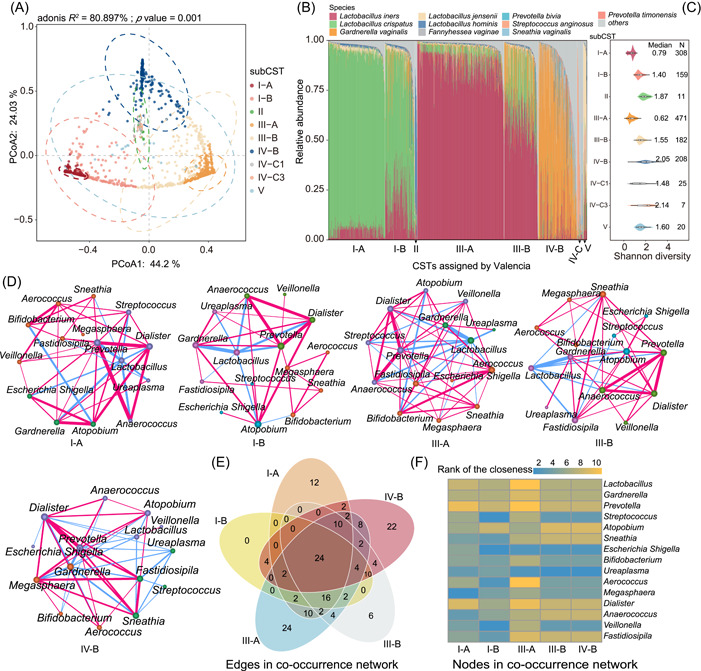
Diversity and composition of the vaginal microbiota in reproductive‐age women. (A) Principal coordinates analysis (PCoA) of the 1391 samples based on unweighted UniFrac distances at the genus level according to community types of the vaginal microbiota. (B) Species composition of all retained samples (*n* = 1391) categorized by community state types (CST) assignment according to VALENCIA. CST I‐A almost completely *Lactobacillus crispatus*; CST I‐B less *L. crispatus* but still majority; CST II communities are dominated by *Lactobacillus gasseri*; CST III‐A almost completely *Lactobacillus iners*; CST III‐B less *L. iners* but still majority; CST IV‐B contains a high to moderate relative abundance of *Gardnerella vaginalis* and *Atopobium vaginae*; CST IV‐C1 dominated by *Streptococcus* spp.; CST IV‐C3 dominated by *Bifidobacterium* spp.; CST V communities are dominated by *Lactobacillus jensenii*. (C) Violon plot of alpha diversity based on the Shannon index. (D) Co‐occurrence bacterial networks in each sub‐CST sample at the species level. Each network was created by computing the co‐occurring bacteria with significant Pearson correlation coefficients. Node properties: (i) circle size, proportional to the normalized and standardized bacterial relative abundances; (ii) color, communities as retrieved by the Louvain algorithm. Edge properties: (i) thickness, proportional to *p*‐value of the Pearson correlation coefficient, from the most significant (thicker) to the less significant (thinner); (ii) color, red for positive and green for negative Pearson correlation coefficients. (E) The number of unique and shared edges among five CSTs. (F) The centralities (rank of the closeness) and discrepancies of nodes in five CST co‐occurrence networks.

To further understand the variation in microbiota communities present in the vagina, we examined community state types (CST) across all samples using the VALENCIA, a nearest centroid‐based classifier [16]. Based on their similarity to a set of reference centroids, we found that all blank controls have lower similarity scores (all less than 0.3). Thus, we retained these vaginal samples with similarity scores greater than 0.3 (1391 samples) for further analysis (Table S5). This analysis revealed five major groups and nine subgroups of microbial communities (Figure 2A), which are reminiscent of the groups identified in previously published studies on microbial diversity in the human vagina [17, 18]. The five community types here designated as I, II, III, IV, and V, contain 467, 11, 653, 240, and 20 samples, respectively. The taxonomic composition at the species level of samples designated to each CST is typically aligned with that of the related reference centroid (Figure 2B). As anticipated, variations in Shannon diversity were noted among the sub‐CSTs, including I‐A (*n* = 308), I‐B (159), II (11), III‐A (471), III‐B (182), IV‐B (208), IV‐C1 (25), IV‐C3 (7), and V (20). The groups I‐A and III‐A showed a lower Shannon index (Figure 2C and Table S6). To investigate the concordance of microbial co‐occurrence of different CSTs, we next computed the correlations between bacterial species in each CST. The co‐occurring vaginal bacterial networks showed several differences between the five community types with samples of more than one hundred and eighty: (i) the CST I‐A, III‐A, and IV‐B microbiota had two linked communities, while the CST I‐B and III‐B microbiota had four and three linked communities, respectively (Figure 2D). Sixteen edges were shared among five CSTs (Figure 2E); (ii) the closeness and eigenvector of shared nodes among five CSTs were also quite different (Figure 2F), III‐A and III‐B have 10 and 12 unique edges, respectively (Figure 2E); (iii) *L. iners* was mostly positive correlated with other species in CST I‐A and IV‐B, and negatively correlated to most bacteria in other three CSTs. At the genus level, *Lactobacillus* exhibited both positive and negative connections within the IV‐B microbiota while showing only negative relations in the other four CST microbiota, *Gardnerella* was mostly negatively correlated with the other genus and positively correlated to most bacteria in the other four CSTs (Figure S2).

### Association of the vaginal microbiota with biochemical and clinical measurements

**Figure 3:**
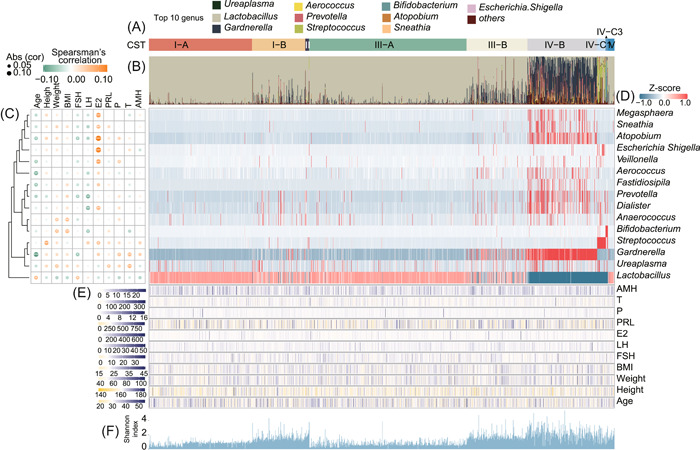
Heatmap of the abundances of microbial taxa significantly correlated with four biochemical and seven clinical observations from 1391 women of reproductive age. (A) Vaginal samples were divided into nine subtypes. (B) The samples based on the abundances of the top 10 genera of vaginal bacterial communities are shown. (C) Complete linkage clustering of taxa based on Spearman's correlation coefficient profiles, which were defined as the set of Spearman's correlation coefficients calculated between the genus composition and the measurement scores of a sample. Yellow tiles indicate positive associations between these measurements and genera; green tiles indicate negative associations. *, **, and *** represent significant differences at *p* < 0.05, *p* < 0.01, and *p* < 0.001, respectively; abs (cor) represent the absolute value of Spearman's correlation coefficient. (The color key is indicated in the upper left corner). (D) Heatmap of the abundances of 15 genera from 1391 women of reproductive age (color key is indicated in the upper right corner). (E) Four biochemical and seven clinical observations for each of the 1391 samples. (F) Shannon diversity indices were calculated for 1391 vaginal samples.

The CSTs were presented according to vaginal microbiota composition at the genus level (Figure 3A,B). To explore the relationship between vaginal microbiota and the biochemical and clinical measurements, we calculated the correlations between the relative abundance of the vaginal microbiota (Table S7) and 11 measurements at the genus level using Spearman's correlation analysis (Table S8). Only a small number of microbes had a significant correlation (*p* < 0.05) with one or more measurements. Estradiol (E2) was found to be significantly associated with the largest number of microorganisms (Figures 3C and S3A). The heatmap shows the taxon abundance of the genus correlated with clinical or biochemical measurements (Figure 3D). In women of reproductive age, the relative abundances of *Lactobacillus* were found to be positively correlated with age (*p* < 0.05), suggesting an increase in the colonization of these bacteria in the vagina as females age. In addition, the relative abundances of *Dialister, Prevotella*, and *Bifidobacterium* were negatively correlated (*p* < 0.05) with LH. The relative abundance of *Veillonella*, *Dialister*, *Escherichia Shigella*, *Atopobium*, *Sneathia*, *Megasphaera*, and *Streptococcus* were positively correlated with E2 (Figure 3C,D). The distributions of 11 measurements and alpha diversity values were also plotted (Figure 3E). Similarly, at the species level, the relative abundances of *L. jensenii*, *Streptococcus agalactiae*, *Lactobacillus sp*., *Lactobacillus gasseri*, *Fannyhessea vaginae*, *Escherichia coli*, and *Veillonella montpellierensis* were positively correlated with E2 (*p* < 0.05) (Figure S3B). These results were confirmed by Linear discriminant analysis Effect Size (LEfSe) analysis between the bottom and top 50% of samples for each measurement (Figure S4). For example, *Lactobacillus* was a biomarker for age, and *G. vaginalis* was a biomarker for weight. The distributions of alpha diversity values were also plotted (Figure 3F).

### Differences in the vaginal microbiomes of women suffering various types of infertility

For all 1411 samples, we recorded whether the patients were diagnosed with any symptoms (PCOS, premature ovarian insufficiency, chronic salpingitis, endometritis, pelvic inflammatory disease [PID], endometriosis, intrauterine adhesion, scarred uterus, ovulation failure, hydrosalpinx, fallopian tube obstruction, or salpingectomy). The 180 samples from patients without any female infertility symptoms and with a sex partner diagnosed with azoospermia, oligozoospermia, asthenospermia, or sperm deformity were considered the “male factor” group (control group). Among the 1231 women with infertility, most of them (783, 63.61%) were diagnosed with an inflammation condition, namely chronic salpingitis, endometritis, and pelvic inflammatory disease (Figure S5A). In addition, 976 (69.17%) of them had tubal infertility conditions (including chronic salpingitis, fallopian tube obstruction, and hydrosalpinx), and 758 of these patients were diagnosed with chronic salpingitis. Furthermore, 423 (34.36%) patients were diagnosed with at least two conditions. For these 11 clinical and biochemical measurements, women with PCOS, endometriosis, and lower AMH levels showed the highest number of differential measurements compared with the control (Table S1). Next, LEfSe analysis was conducted between the control and each infertility group. Only 12 and 7 infertility‐associated bacterial taxa were identified in females with low AMH and intrauterine adhesion, respectively. Of note, *Lactobacillus* showed higher abundance in women with premature ovarian insufficiency. *Escherichia* was found to be associated with PCOS (Figure S5B). These results indicated that there are some differences in vaginal microbiome composition among reproductive‐age women with different infertility symptoms. The distribution of different community types in each infertility group was also explored. Type III‐A samples showed a high‐level distribution of scarred uterus (*n* = 81). The highest proportion of type IV‐B samples was from women who were diagnosed with salpingectomy (Figure S6).

### Association of vaginal microbiota composition with IVF outcomes

**Figure 4:**
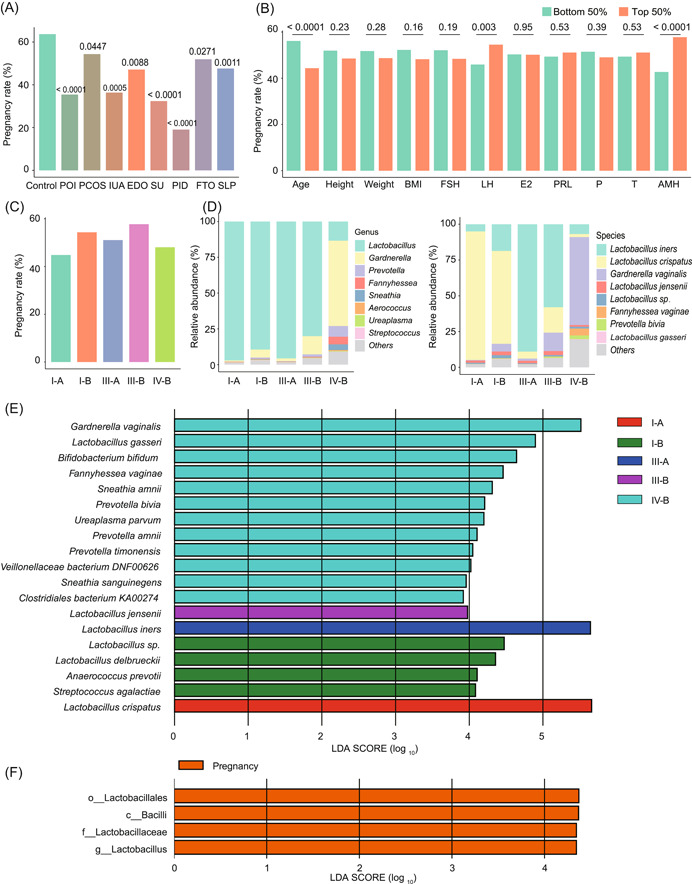
Association of vaginal microbiota composition with in vitro fertilization (IVF) outcomes. (A) Analysis of women who became pregnant in each infertility group compared with control, some women were diagnosed with more than one symptom would be used to compute multiple times. The *p* value of each group compared to the control was presented in the column. (B) Analysis of pregnancy rate in top and bottom 50% sample for each measurement. Chi‐square test was used to compare IVF outcomes between each group and the control. The *p* values were presented above the columns of each measurement. (C) The distribution of pregnancy results for each community state type (CST). (D) The composition of the vaginal microbiome for each type at the genus (left panel) and species level (right panel). (E) Associations between five CSTs and bacterial taxa at the species level were identified by Linear discriminant analysis Effect Size (LEfSe) analysis. (F) Associations between the pregnant and nonpregnant women in CST IV‐B and bacterial taxa were identified by LEfSe. BMI, body mass index; c_, class; E2, estradiol; f_, family; g_, genus; T, testosterone; o_, order; P, progesterone.

Of the 1391 women, 1236 underwent IVF or IVF‐ICSI (Intracytoplasmic sperm injection) with the transfer of fresh or frozen embryos on Day 3. Among these women, 654 (52.91%) of them were found to be human chorionic gonadotrophin (hCG) positive 14 days after the embryo transfer (ET), and 621 (50.24%) of them had a heartbeat detected 35 days after ET (these women were considered pregnant). The remaining 615 women were considered nonpregnant. In addition, the control (65.61%), PCOS (58.39%), and FTO (54.81%) groups showed a higher rate of hCG positive after IVF or IVF‐ICSI (Figure 4A). For the 11 biochemical and clinical measurements, we also analyzed their association with hCG positive rate and pregnancy rate (Figures 4B and S7). Only age, AMH, and LH had a significant effect (*p* < 0.05) on the pregnancy rate.

We next analyzed the bacterial community types within each group of women. Type I and type III contained the largest number of women, 408 (33.01%) and 589 (47.65%), respectively. The proportion of women who became hCG positive and pregnant was lower for the I‐A and III‐A subtypes compared with I‐B and III‐B (type I‐A, 44.81%; type III‐A, 51.07%; type I‐B, 54.35%; type III‐B, 57.74%). Type IV‐B contained 183 women, 88 (48.09%) of whom became pregnant (Figure 4C and Table S9). The samples in CSTs I‐A, I‐B, III‐A, III‐B, and IV‐B were dominated by *Lactobacillus* 97.04%, 89.33%, 95.61%, 79.87%, and 13.38%, respectively (Figure 4D). *L. jensenii*, which is a biomarker for this CST, showed the highest pregnancy rate (Figure 4E). Surprisingly, there was a higher abundance of *Gardnerella* in IV‐B, but this CST did not present a lower pregnancy rate. When we checked the difference between pregnant and nonpregnant women in this CST, we found that the Lactobacillus was a biomarker for the pregnant women (Figure 4F).

### Schematic infertility vaginal microbiome from a metagenome perspective

**Figure 5:**
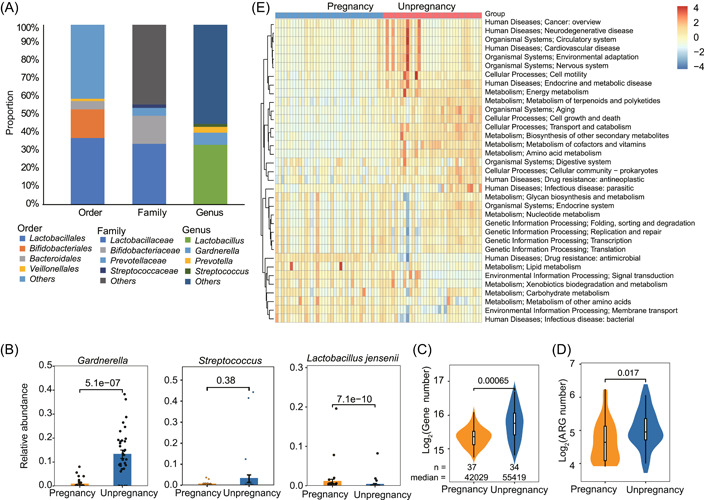
Composition and function difference of vaginal metagenome between pregnant and nonpregnant women. (A) Composition of the vaginal microbiota at different levels (namely order, family, and genus) according to the median relative abundance. The top four taxa were presented, and the left microbiota was combined and labeled as “others.” (B) Relative abundance of the top two genera and one species in pregnant and nonpregnant women. Number of total annotated genes (C) and antimicrobial resistance genes (ARG) genes (D) in two groups of women. (E) The relative abundances of functionally and phylogenetically annotated orthology classified based on the (Kyoto Encyclopedia of Genes and Genomes) KEGG database.

The microbiome, given its crucial role in human health and disease, has significantly revolutionized modern biology [19]. We further performed shotgun metagenomic sequencing of the genomic DNA of 71 vaginal samples. After metagenome assembly and taxonomy annotation, our results showed that the majority of the contigs were assigned to Lactobacillaceae (~33.78% of relative abundance), Bifidobacteriaceae (~15.78%), and Prevotellaceae (~4.31%). At the genus level, the same as 16S rRNA sequencing results, the vaginal flora was also dominated by *Lactobacillus*, followed by *Gardnerella* and *Prevotella* (Figure 5A). Only *Lactobacillus* had a significantly higher abundance in pregnant women compared to nonpregnant women (*p* < 0.05). On the other hand, *Gardnerella* showed a significantly higher abundance in nonpregnant women (*p* < 0.05) (Figure 5B). At the species level, *L. jensenii* exhibited a relatively higher abundance in pregnant women (*p* < 0.05), which is consistent with previous 16sRNA results (Figure 4E) that *L. jensenii* was a marker for CST III‐B with the highest pregnancy rate. Compared to the pregnant women, the nonpregnant women contained more annotated genes (Figure 5C) and antimicrobial resistance genes (ARGs) than pregnant women (Figures 5D, S8, and Table S10). The three genes with the largest prevalence of antibiotic resistance ontology (ARO) in nonpregnant women were *tetM*, *rpoB*, and *vanT*, which are associated with the drug class of tetracycline, rifampicin, and vancomycin antibiotic. In comparison, *qacJ*, *tetM*, and *ErmB* were the largest prevalence genes that are associated with the drug class of quarternary ammonium compounds, tetracycline, and macrolide antibiotics in pregnant women, respectively. According to the functional annotation and abundance information of all genes in Carbohydrate‐Active enzymes (CAZy), evolutionary genealogy of genes: Non‐supervised Orthologous (eggNOG), and Kyoto Encyclopedia of Genes and Genomes (KEGG) databases, the pathways encoded by the microbial genes compared between the two groups (Figure S8), which are closely related to starch degradation [20]. The KEGG pathways related to Human Diseases, including “Drug resistance: antineoplastic” and “Infectious disease: parasitic” of the nonpregnant group, were significantly greater than that of pregnant women (Figure 5E). While “Membrane transport,” and “Infectious disease: bacterial” pathways trended to be higher in pregnant women.

## DISCUSSION

### Community types and function of vaginal microbiota in females with infertility

We characterized the vaginal microbiota of 1411 sexually active women who were diagnosed with infertility. The existence of five distinct vaginal bacterial communities in females with different infertility symptoms is consistent with previous studies showing that there are five community types in the vagina [18, 21]. The higher abundance of *Gardnerella* and lower abundance of *Lactobacillus* in community type IV might correspond to a more diverse vaginal microbiota, as reported previously [22, 23]. Variations in the vaginal microbiota communities among individuals could be attributed to both the innate and adaptive immune systems. Additionally, the composition and quantity of vaginal secretions, along with ligands on epithelial cell surfaces, may play a pivotal role in shaping the diverse vaginal communities [21, 22]. In addition, human habits, ethnicity, and practices also exert strong influences [24]. Here, the genome‐resolved metagenome analyses were conducted to offer a better exploitation of the metagenomic data to comprehensively understand vaginal microbial function and association with pregnancy. After genome annotation, we find that nonpregnant individuals harbor higher numbers and abundance of antibiotic‐resistance genes compared with these pregnant ones. In addition, metagenome analyses showed that nonpregnant women's microbiota were enriched in “Environmental adaptation,” “Drug resistance: antineoplastic,” and “Infectious disease: parasitic” pathways. These factors may help explain why individuals with a higher number of resistance genes in their vaginal microbiota experienced poorer IVF outcomes. Nonetheless, our study offers a comprehensive overview of antibiotic‐resistance genes in the human vaginal microbiota.

### Correlation profiles of taxa and 11 biochemical and clinical measurements

Afterward, we investigated the correlation profiles between microorganisms and 11 biochemical and clinical measurements. This aligns with a prior study demonstrating that estrogen levels notably impact the composition of the vaginal microbiome [25]; we also detected the most number of microorganisms (seven genera (*[annyhesea*, *Escherichia. Shigella*, *Megasphaera*, *Veillonella*, *Dialister*, *Sneathia*, and *Streptococcus*] and nine species) significantly (*p* < 0.05) correlated with this E2 compared with other hormones. Estrogen, known for promoting the synthesis and storage of glycogen, is found at low levels in girls before puberty and women after menopause. Hence, it is unsurprising that the microbiomes of post‐menopausal individuals comprise a diverse array of anaerobic bacteria. A prior study also noted higher levels of the hormones E2 and progesterone (P) in premenopausal women with a BRCA mutation [26]; the suggestion is that P might impact vaginal epithelial cells, potentially counteracting estrogen and leading to an imbalanced microbiome. Additionally, it was found that gut bacteria could influence estrogen metabolism, thereby affecting vaginal epithelial cells [27]. *Fannyhesea* and *Citrobacter*, which are bacterial vaginosis‐associated genera, have been extensively investigated [28, 29] and found to be positively correlated with E2 in this study. A previous study indicated that *Fannyhesea* was significantly negatively correlated with AMH and positively correlated with FSH and LH [30], which are thought to be important for pathogenesis [31, 32, 33, 34]. For example, *Fannyhesea vaginae* have been strongly associated with bacterial vaginosis (BV), independently by different groups and with biofilm in BV [35]. In addition, microbial composition varies with the timing of the menstrual cycle, so some markers are not always consistent with previous studies. More studies with larger sample sizes and menstrual cycles should be carried out in the future.

### Comparison of the vaginal microbiota between pregnant and nonpregnant women

The primary bacteria at the vaginal site belong to the genus *Lactobacillus*, known for producing lactic acid, which helps maintain an acidic pH in the vagina, acting as a barrier against pathogens [36]. Previous studies have found associations between abnormal vaginal microbiota and poor reproductive outcomes [13, 37]. This suggests that vaginal microbiome profiling facilitates the stratification of pregnancy chances before commencing IVF or IVF‐ICSI treatment [15]; another study did not find statistically significant differences between pregnancy rates and microbiome composition [38]. Among *Lactobacillus*, *L. iners* is a genuine vaginal symbiont [39]. Numerous studies have reported an association between the presence of *L. crispatus* in the vagina and good health [40]. Here, we found that an extremely higher abundance of *L. iners and L. crispatus* in CST I‐A and CST III‐A (higher than ~90%) might not benefit IVF outcomes (44.81% and 51.06%, respectively). The process through which *Lactobacillus* species in the vagina produce both enantiomers of lactic acid (l‐lactic and d‐lactic acid) remains unclear at present [41]. The dominance of *L. iners* in the vaginal microbiota was found to be associated with low vaginal pH < 4.5. due to its production of l‐lactic acid [42]. d‐Lactic acid levels were found to be higher in samples containing *L. crispatus* compared to those with *L. iners* [43]. Recent research indicates that within the tumor microenvironment, lactic acid bacteria can alter tumor metabolism and lactate signaling pathways, potentially contributing to the development of therapeutic resistance [44]. Furthermore, the genetic makeup of *L. iners* strains includes inerolysin, a pore‐forming toxin that shares similarities with vaginolysin found in *G. vaginalis* [45]. The negative effect of these lactic acid bacteria and accumulated toxins could be promising therapeutic targets across cancer types and poor IVF outcomes.

It is noted that type IV‐B patients had a comparable pregnancy rate (48.09%) with type I‐A (44.81%) and type III‐A (51.07%). This finding was surprising because this community type is dominated by the genus *Gardnerella* and species *G. vaginalis* (61.38%). A recent study found that the extreme heterogeneity makes it improbable to discover either a universal pathogen for BV (diagnostic OTUs or their cultures) or a universal dysbiotic BV state [46]. *Lactobacillus* was not dominant or even absent in the vaginal flora in some women, but these women did not report symptoms of BV [47]. This explained the normal pregnancy rate of CST IV‐B. Moreover, a meta‐analysis of 12 studies in an IVF setting concluded that bacterial vaginosis (BV) did not significantly impact the live birth rate [48]. There is evidence that *Gardnerella* can encourage the colonization and virulence of other human pathogens and that sialidase could be an important factor [49].

## CONCLUSION

In conclusion, previous studies have suggested associations between the microbiome composition of the female reproductive tract and reproductive outcomes in infertile women undergoing assisted reproduction. Our aim was to determine whether the vaginal microbiota composition is associated with reproductive outcomes of five designated community types, eleven biochemical and clinical measurements. We used 16S rRNA gene sequencing to analyze vaginal samples in a cohort of 1411 infertile patients and then 1255 patients undergoing assisted reproductive treatments. The results indicated that a too‐high or too‐low abundance of *Lactobacillus* species has a negative impact on reproductive health, suggesting that a moderate level of *Lactobacillus* species (*L. iners and L. crispatus*) was beneficial for IVF outcome. These findings may aid the development of strategies for predicting vaginal microbiota to control IVF outcomes.

## METHODS

### Patients and samples

A total of 1411 patients diagnosed with infertility (which is defined as the inability to conceive after a year of regular unprotected sexual activity) were used in this study. Detailed medical information was collected from patients, including age, height, body weight, and BMI. Diagnoses include endometriosis, tubal factor, ovulatory disorder, unexplained infertility, and/or male factors. Individuals were excluded from the study due to prescription of antibiotics within a month before sample collection; uncontrolled endocrine disorders such as hyperthyroidism, diabetes mellitus, hypothyroidism, and hyperprolactinemia; current sexually transmitted or infectious diseases; a history of ART; mental diseases; uterine malformations; hereditary diseases; smoking; alcoholic consumption; gynecological malignancy; and severe systemic diseases that affect maternal health. Among them, the 180 diagnosed with male factors that led to infertility were used as the control group in this study. All vaginal samples were obtained from the posterior fornix during speculum examination (from January 2022 to June 2022) during the natural menstrual cycle Days 15–18. Samples were immediately frozen at −80°C until further use. From July 2022 to May 2023, 34 patients who did not get pregnant after ≥3 times of IVF/ICSI were further metagenome sequenced. Meanwhile, 37 patients who got pregnant at the first IVF/ICSI cycle were randomly selected as control.

### Biochemical measurements

On the natural menstrual cycle Days 2–3, venous blood samples were taken between 8 a.m. and 9 a.m. after a 12‐h overnight fast. Serum hormone levels such as FSH, LH, PRL, E2, total testosterone (TT), AMH, and P, were measured with chemiluminescence immunoassays COULTER DXI 800 (Beckman).

### Reproductive outcome analysis

Among the total 1411 patients, 1255 received fresh (frozen) cleavage‐stage (Day 3) or blastocyst (Day 5) ET (embryo transplantation) in the first IVF/ICSI cycle. All patients were first‐time to conduct ET. Following ET, the study recorded the biochemical pregnancy rate (positive hCG at Day 14) and the clinical pregnancy rate (ultrasound‐proven fetal heartbeat at 7 weeks of gestation). Those who achieved clinical pregnancy were assigned to the pregnant group, while the others were assigned to the nonpregnant group for each infertility group.

### Microbial genomic DNA extraction, 16S rRNA amplicon, and sequencing

Genomic DNA was extracted from vaginal swabs (*n* = 1411) along with nontemplate negative controls to address potential background DNA contamination (*n* = 25). Total microbial genomic DNA extraction was performed using the Ezup Oral Swabs Genomic DNA Extraction Kit (Sangon Biotech) following the manufacturer's instructions. The V3–V4 hypervariable region of the bacterial 16S rRNA was amplified using universal primers 341F: CCTACGGGNGGCWGCAG; 805R: GACTACHVGGGTATCTAATCC [50]. The 16S ribosomal RNA genes were sequenced with the Illumina MiSeq platform to generate 300‐bp pair‐end reads at Sangon Biotech Co., Ltd.

### 16S rRNA‐seq data processing, denoise, and species annotation

The raw data obtained by sequencing were first quality filtered: PhiX sequences were removed, and paired‐end reads with *Q* scores ≥20 were joined using QIIME2 software [51] and EasyAmplicon [52]. The DADA2 method [53] is used for noise reduction. It performs dereplication or equivalent to 100% similarity clustering. Each de‐duplicated sequence generated after noise reduction using DADA2 is called ASVs (Amplicon Sequence Variants) corresponding to the OTU representative sequence. By applying QIIME2's classify‐sklearn algorithm, a pretrained Naive Bayes classifier is used for species annotation of each ASV. Based on the ASVs annotations and the feature tables of each sample, taxa abundance tables were obtained at the levels of kingdom, phyla, class, order, family, genus, and species. When considered taxa present in at least 5% of individuals in our cohort, that left 16/35 (remaining/total) phyla, 22/28 classes, 52/137 orders, 91/215 families, 154/446 genera, and 322/1550 ASVs. Genera not colonizing humans or associated with kitome contaminants were removed from further analysis [54]. In our study of vaginal 16S rRNA profiles, we employed a simple statistical method to identify and remove potential contaminant sequences in marker‐gene and metagenomics data [55]. Subsequently, the abundance of each taxon was analyzed, leading to the removal of low‐abundance species from further consideration. We retained taxa that exhibited an abundance of at least 1% in at least 15% of samples. To obtain specific species, the assignments of the retained ASVs were searched based on BLASTn to annotate species. The 100% percentage of identity and expectation value was considered to select significant BLAST hits. CSTs were assigned using VALENCIA, a nearest centroid‐based classifier [16].

### Alpha and beta diversity

Alpha diversity indicates the number of species present within a typical area or ecosystem. Here, alpha diversity was used to analyze the complexity of species richness and diversity for a sample based on normalized ASV abundances calculated with the Shannon index using the QIIME2 software [51]. Principal coordinates analysis (PCoA) of relative abundance profiles for all 1411 samples at the genus level was conducted. The dimension ordination provided position values along an ordination axis and distances from the axis for samples of communities. The data were visualized by ImageGP [56].

### Co‐occurrence analysis

For the co‐occurrence network analysis, the bacterial correlations in CST I‐A, I‐B, III‐A, III‐B, and IV‐B were computed based on the relative abundance of each genus using SparCC with 100 bootstraps to estimate the *p* value [57, 58]. The correlation values with a *p* < 0.05 were retained. The co‐occurrence networks of the five vaginal microbiota communities were visualized using Gephi software (https://gephi.org/). The closeness and eigenvector of the nodes were calculated to measure node centrality in each network [59].

### Between‐group variation analysis

High‐dimensional biomarkers were discovered by LEfSe [60] using the parameter “linear discriminant analysis (LDA) score > 4” to identify characteristics of abundance and related classes (e.g., genes, metabolites, or taxa). LEfSe (Linear discriminant analysis Effect Size) identifies different features (such as ASVs, genus, or genes) most likely to explain differences between classes by combining standard tests for statistical significance with additional tests encoding biological consistency and effect relevance.

### Identification of microbiome composition related to clinical and biochemical factors

Age, height, body weight, body mass index, and seven hormone levels of the 1411 individuals were then compared with the microbiome composition. The Spearman's *r* between the 11 factors and the abundance of each microbe at genus and species levels. A significant correlation between the presence of a microorganism and the clinical and biochemical measurements was considered if *p* < 0.05, as determined using the psych R package (version 4.1.2) with the *p* value adjusted using the Benjamini–Hochberg method.

### Metagenome sequencing

Seventy‐one vaginal samples were collected (Table S3) for metagenomic sequencing. After conducting library quality control, the different libraries were pooled based on their effective concentration and the desired data amount. The 5′‐end of each library was phosphorylated and cyclized. Subsequently, loop amplification was performed to generate DNA nanoballs. Finally, these DNA nanoballs were loaded into a flow cell with DNBSEQ‐T7 for sequencing at Novogene Bioinformatics Technology Co., Ltd.

### Metagenomic data analysis

The data quality control process involved discarding paired reads if (a) Either read contained adapter contamination, (b) More than 10% of bases were uncertain in either read, and (c) The proportion of low‐quality bases (Phred quality <5) exceeded 50% in either read. This process was carried out to obtain clean data using Fastp (version 0.23.1). [61]. Samples passing QC were assembled initially by MEGAHIT (v1.0.4) (‐‐presets meta‐large) [62], and SOAP denovo [63] was used to conduct assembly analysis. The assembled Scaffolds are broken at the N junction to produce N‐free sequence fragments, which are called Scaftigs [64]. Then clean data of all samples were mapped to assembled Scaftigs using Bowtie2 (Version: 2.2.4) [65], and unutilized PE reads were collected. The effective Scaftigs, with a length of 500 bp or more, were utilized for further analysis and gene prediction [64, 66]. These Scaftigs were employed for Open Reading Frame (ORF) prediction using MetaGeneMark (Version 2.10) [67].

The ORFs that were less than 100 nucleotides in length were trimmed. Subsequently, the ORFs were dereplicated using CD‐HIT [68] to generate gene catalogs. In this context, the nonredundant continuous gene encoding the nucleic acid sequence is referred to as genes [69]. Dereplicating by default: identity = 95%, coverage = 90%. The longest gene was selected as the representative gene, also known as the unigene. CD‐HIT parameter [68]: ‐c 0.95, ‐G 0, ‐aS 0.9, ‐g 1, ‐d 0; Clean data were mapped to gene catalog using Bowtie2 to calculate the quantity with parameter: ‐‐end‐to‐end, ‐‐sensitive, ‐I 200, ‐X 400. The gene abundance was determined based on the total number of mapped reads and the length of the gene [70, 71]. Downstream analyses were conducted using the abundance of gene catalogs. Subsequently, we utilized sequence or phylogenetic similarity to the database sequences (microNR database) to taxonomically annotate each metagenomic homolog (MEGAN) [72, 73]. The abundance tables of different taxonomic ranks were based on the gene abundance table. After quality control of the data, we obtained a total of 5.12 terabytes (Tb) of clean data (with an average of ~72.42 Gb per sample) (Table S3). Then, to decrease potential DNA contamination from the host, we mapped the sequence data to the human genome, and 364.31 Gb (with an average depth of ~5.20 Gb per sample) of sequence data remained for the subsequent analyses.

### Function annotation

The functional diversity of a community was quantified by annotating metagenomic sequences with functions, requiring at least one High‐scoring Segment Pair (HSP) with a score exceeding 60 bits [60]. Protein coding sequences were aligned or mapped against functional databases, including KEGG [74], evolutionary genealogy of genes: Nonsupervised Orthologous Groups (eggnog, Version: 5.0) [75], and Carbohydrate‐Active enzymes Database (CAZy, Version: 2023.03) [76].

### ARGs analysis

CARD (the Comprehensive Antibiotic Research Database) database [77] is used to facilitate the identification and characterization of antibiotic‐resistance genes. All unique genes were blastp against the CARD database (blastp, e‐value ≤ 1e−5) [78]. Each gene or type is annotated with comprehensive information, encompassing resistance profiles and ARO mechanisms [79].

## AUTHOR CONTRIBUTIONS

Tao Wang, Penghao Li, and Diyan Li wrote the paper. Xue Bai, Maosheng Yang, Dong Leng, and Shilin Tian analyzed the data, prepared figures. Hua Kui, Sujuan Zhang, Xiaomiao Yan, Qu Zheng, Pulin Luo, Changming He, and Yan Jia collected biological samples. Hua Kui, Sujuan Zhang, and Huimin Qiu did the experiments. Zhoulin Wu and Rurong Mao provided materials and equipment. Jing Li, Feng Wan, and Muhammad Akhtar Ali revised the manuscript. Shilin Tian and Yong‐Xin Liu designed the bioinformatics analysis process. Diyan Li, Yong‐Xin Liu, and Rurong Mao supervised this project. All authors have read the final manuscript and approved it for publication.

## CONFLICT OF INTEREST STATEMENT

The authors declare no conflict of interest.

## ETHICS STATEMENT

The ethics application (No. 014) was approved by the Ethical Committee of Chengdu Xi Nan Gynecological Hospital. All patients signed written informed consent.

## Supporting information


**Figure S1**: Spearman correlations among different species.
**Figure S2**: Co‐occurrence bacterial networks of the vaginal microbiota in reproductive‐age women.
**Figure S3**: Heatmap of the abundances of microbial taxa at species level correlated with four biochemical and seven clinical observations from 1391 women of reproductive age.
**Figure S4**: Barplot showing clinical observations associated with bacterial taxa at genus (A) and species (B) level identified by LEfSe.
**Figure S5**: Characteristics of each type of infertility.
**Figure S6**: Representation of vaginal bacterial community types (I‐A, I‐B, III‐A, III‐B, IV‐B) within each group of women.
**Figure S7**: Association of vaginal microbiota composition with in vitro fertilization (IVF) outcomes.
**Figure S8**: Function difference of vaginal metagenome between pregnant and non‐pregnant women.

**Table S1**: Biochemical and clinical measurements of each individual.
**Table S2**: Sequencing data summary.
**Table S3**: List of OTUs present in more than 15% of samples.
**Table S4**: Association of the vaginal microbiota with biochemical and clinical measurements.
**Table S5**: Metagenome data summary of 71 samples.
**Table S6**: Alfa diversity indexes of each sample.
**Table S7**: Relative abundance at the genus level of each sample.
**Table S8**: Association of the vaginal microbiota with biochemical and clinical measurements.
**Table S9**: Pregnancy rate of each CST.
**Table S10**: ARG number in each sample.

## Data Availability

All the sequencing data have been deposited in the Genome Sequence Archive (GSA, https://ngdc.cncb.ac.cn/gsa) with accession number: HRA002649 and in the Sequence Read Archive (accession code PRJNA908820 and PRJNA908815). The data and scripts used are available on GitHub (https://github.com/Xuebai2819/Human_Vaginal_microbiome_code). Supplementary materials (figures, tables, scripts, graphical abstract, slides, videos, Chinese translated version, and updated materials) may be found in the online DOI or iMeta Science http://www.imeta.science/.
