# Supplementary material for: Vaginal microbiota are associated with in vitro fertilization during female infertility
Source: Imeta. 2024 Mar 19;3(3):e185. doi: 10.1002/imt2.185 (PMC11183179; doi:10.1002/imt2.185)
Supplement: Supplementary file 1 — Figure S1: Spearman correlations among different species. Figure S2: Co‐occurrence bacterial networks of the vaginal microbiota in reproductive‐age women. Figure S3: Heatmap of the abundances of microbial taxa at species level correlated with four biochemical and seven clinical observations from 1391 women of reproductive age. Figure S4: Barplot showing clinical observations associated with bacterial taxa at genus (A) and species (B) level identified by LEfSe. Figure S5: Characteristics of each type of infertility. Figure S6: Representation of vaginal bacterial community types (I‐A, I‐B, III‐A, III‐B, IV‐B) within each group of women. Figure S7: Association of vaginal microbiota composition with in vitro fertilization (IVF) outcomes. Figure S8: Function difference of vaginal metagenome between pregnant and non‐pregnant women. [file IMT2-3-e185-s002.docx]

**Supporting information to:**

**Vaginal microbiota are associated with in vitro fertilization during female infertility**

**Running title:** Vaginal microbiota in infertile patients

Tao Wang^1#^, Penghao Li^2^^#^, Xue Bai^3,4#^, Shilin Tian^5#^, Maosen Yang^1^, Dong Leng^4^, Hua Kui^4^, Sujuan Zhang^2^, Xiaomiao Yan^2^, Qu Zheng^2^, Pulin Luo^2^, Changming He^2^, Yan Jia^2^, Zhoulin Wu^6^, Huimin Qiu^7^, Jing Li^7^, Feng Wan^8^, Muhammad Akhtar Ali^9^, Rurong Mao^2*^, Yongxin Liu^3*^ and Diyan Li^1*^

^1^Antibiotics Research and Re-evaluation Key Laboratory of Sichuan Province, Sichuan Industrial Institute of Antibiotics, School of Pharmacy, Chengdu University, Chengdu 610106, China.

^2^Jinxin Research Institute for Reproductive Medicine and Genetics, Chengdu Jinjiang Hospital for Maternal and Child Health Care, Chengdu 610000, China

^3^Agricultural Genomics Institute at Shenzhen, Chinese Academy of Agricultural Sciences, Shenzhen 518000, China

^4^College of Animal Science and Technology, Sichuan Agricultural University, Chengdu 611130, China.

^5^College of Life Sciences, Wuhan University, Wuhan 430072, China

^6^College of Food and Biological Engineering, Chengdu University, Chengdu 610106, China

^7^College of Agriculture, Kunming University, Kunming 650214, China

^8^State Key Laboratory of Southwestern Chinese Medicine Resources, Chengdu University of Traditional Chinese Medicine, Chengdu 611137, China

^9^School of Biological Sciences, University of the Punjab, Lahore 54000, Pakistan

^#^These authors contributed equally: Tao Wang, Penghao Li, Xue Bai, Shilin Tian

*Correspondence: [maorr@jxr-fertility.com](mailto:maorr@jxr-fertility.com) (Rurong Mao), [liuyongxin@caas.cn](mailto:liuyongxin@caas.cn) (Yongxin Liu), [lidiyan@cdu.edu.cn](mailto:lidiyan@cdu.edu.cn) (Diyan Li)

**Supplementary figures**


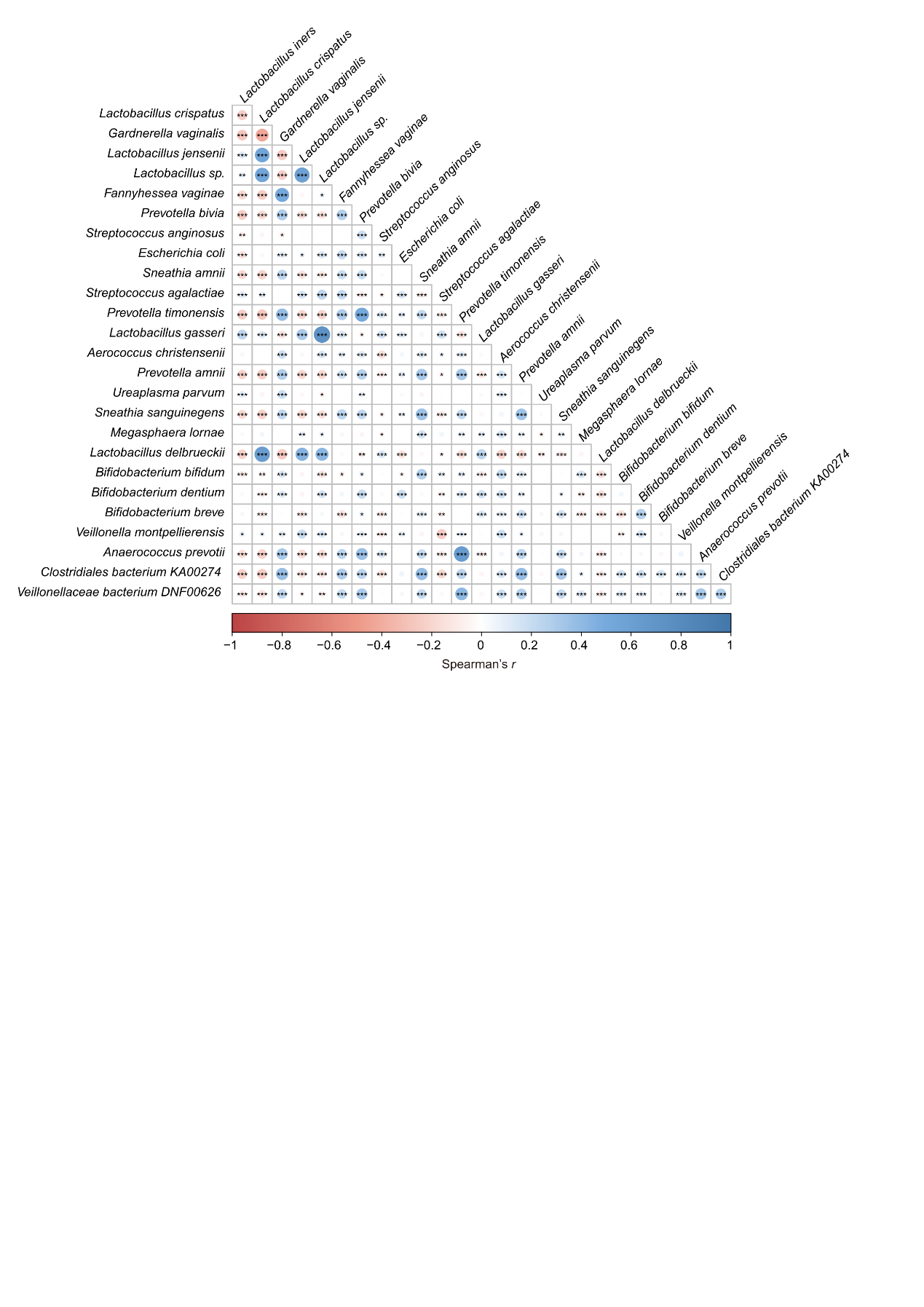


**Figure S1 Spearman correlations among different species.** The size of the circle represents |*r*|, the statistical significance of the *p* values was calculated using a two-sided hypothesis testing. *, ** and *** represent *p* < 0.05, *p* < 0.01, and *p* < 0.001, respectively.


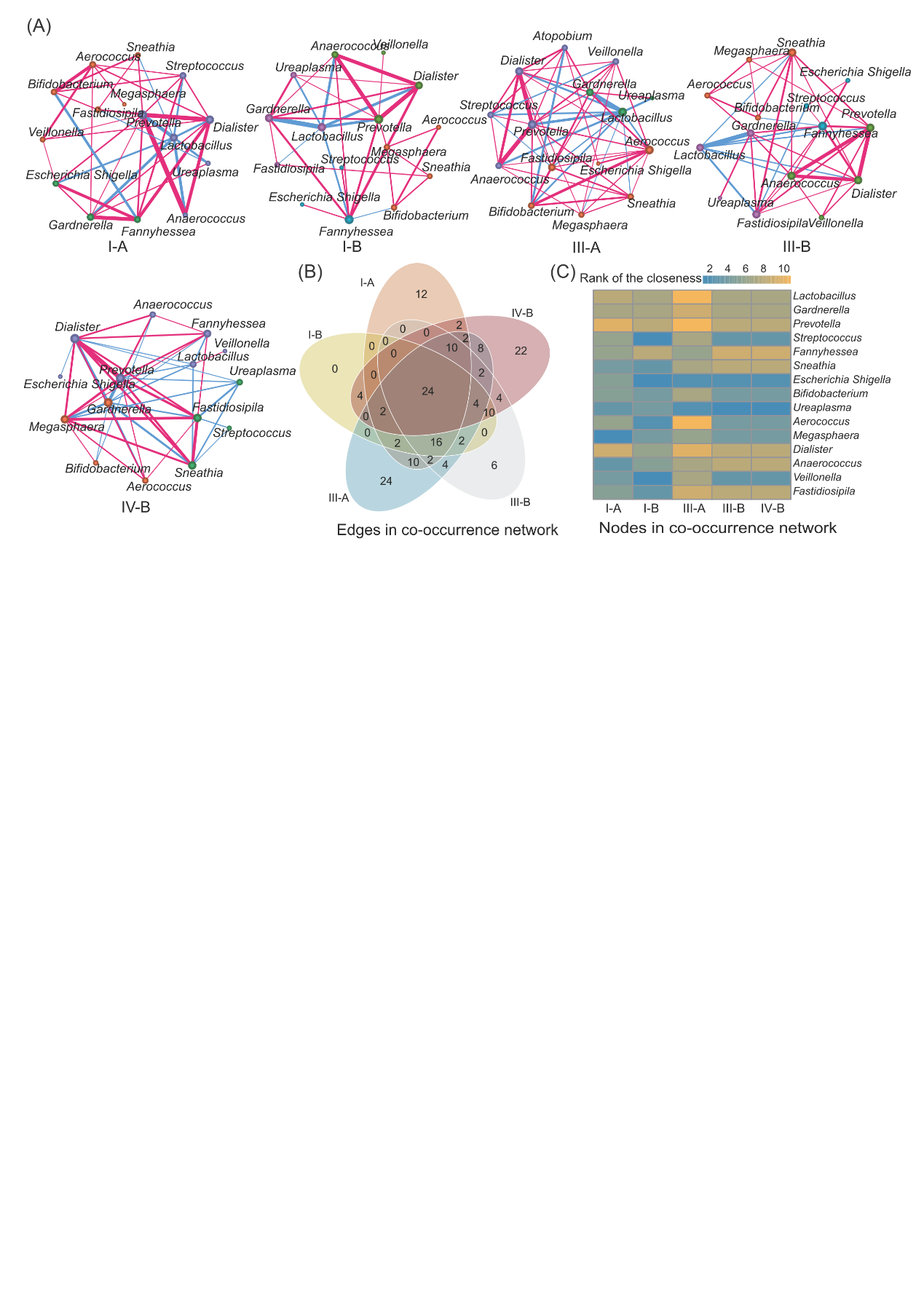


**Figure S2** **Co-occurrence bacterial networks of the vaginal microbiota in reproductive****-age women.** (A) Co-occurrence bacterial network in each sub-CST sample at genus level. Each network was created by computing the co-occurring bacteria with significant Pearson correlation coefficients. Node properties: (i) circle size, proportional to the normalized and standardized bacterial relative abundances; (ii) color, communities as retrieved by the Louvain algorithm. Edge properties: (i) thickness, proportional to *p* value of Pearson correlation coefficient, from the most significant (thicker) to the less significant (thinner); (ii) color, red for negative and grey for positive Pearson correlation coefficients. (B) The number of unique and shared edges among five CSTs. (C) The centralities (rank of the closeness) and discrepancies of nodes in five CST co-occurrence networks.


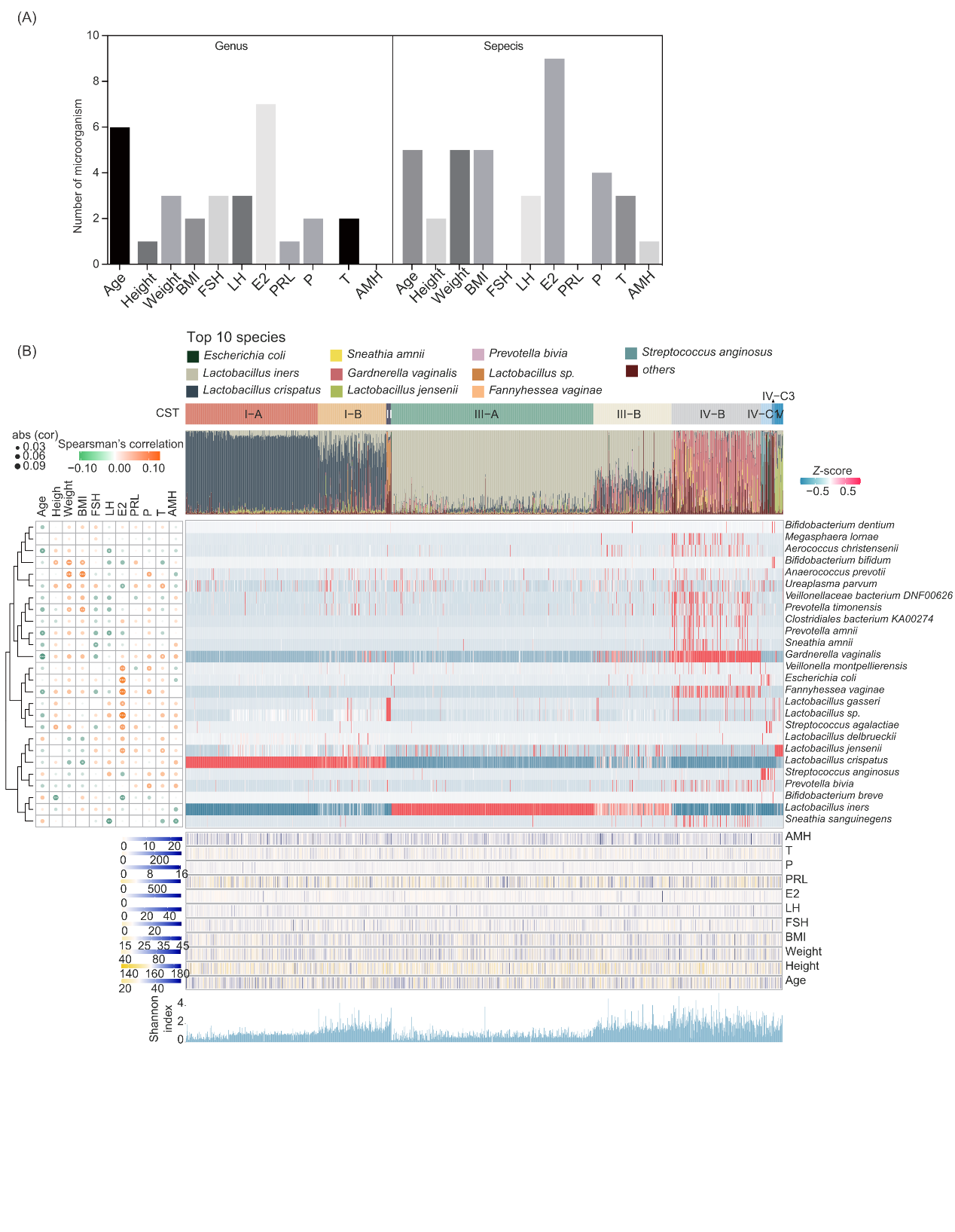


**Figure S3** **Heatmap of the abundances of microbial taxa at species level correlated with four biochemical and seven clinical observations from 1391 women of reproductive age.** (A) The number of microorganisms significantly correlated (*p* < 0.05) with each biochemical and clinical measurement at the genus level. (B) Vaginal samples were divided into nine sub-types. The samples based on the abundance of the top 10 species of vaginal bacterial communities were shown. Complete linkage clustering of taxa based on Spearman’s correlation coefﬁcient proﬁles, which were deﬁned as the set of Spearman’s correlation coefﬁcients calculated between the species composition and the measurement scores of a sample. Yellow tiles indicate positive associations between these measurements and genera; green tiles indicate negative associations. *, **, and *** represent signiﬁcant differences at *p* < 0.05, *p* < 0.01, and *p* < 0.001 respectively; abs (cor) represent the absolute value of the Spearman’s correlation coefﬁcient. (The color key is indicated in the upper left corner). Heatmap of the abundances of 26 species from 1391 women of reproductive age (color key is indicated in the upper right corner). Four biochemical and seven clinical observations for each of the 1391 samples. Shannon diversity indices were calculated for 1391 vaginal samples.


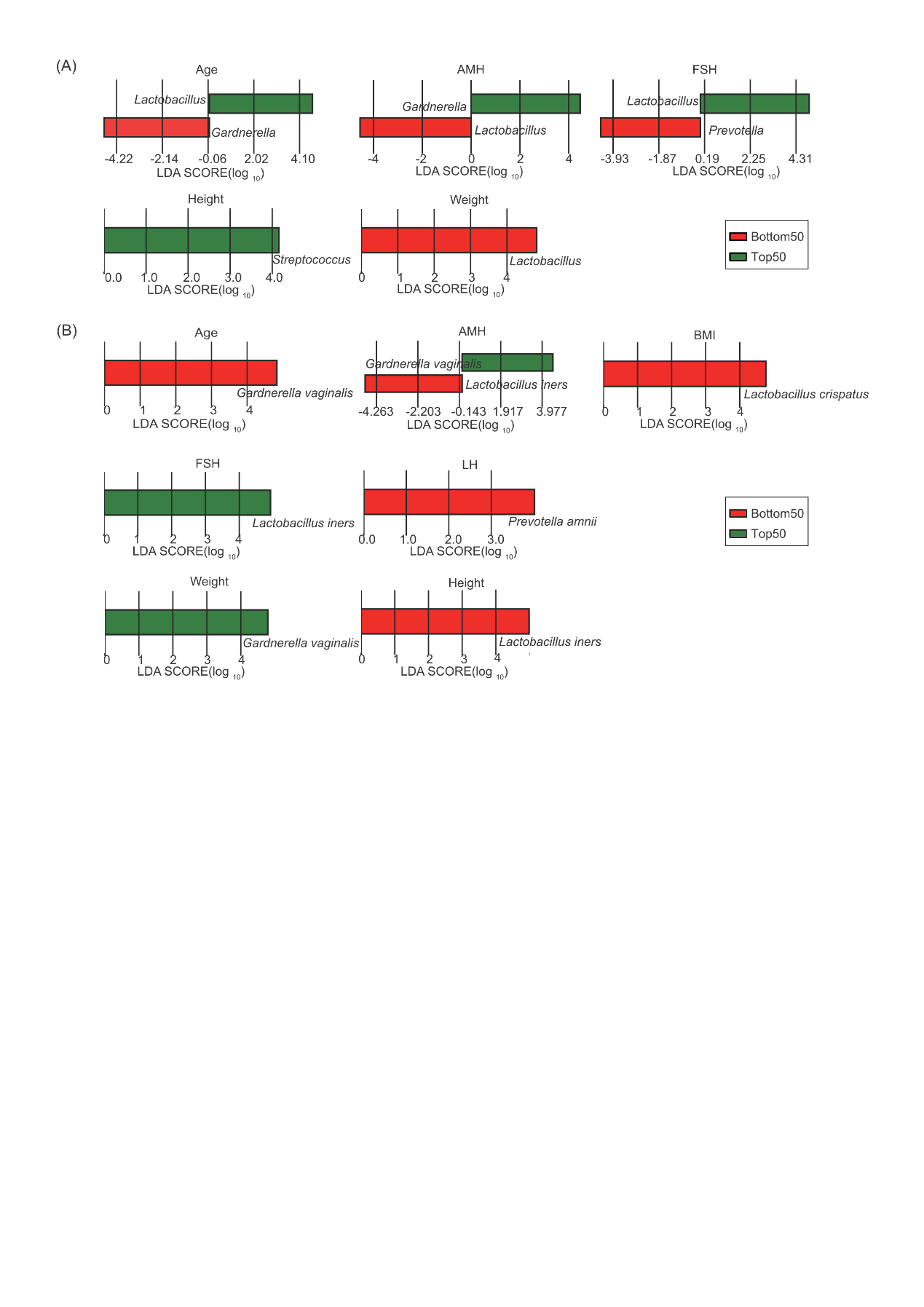


**Figure S4 Barplot showing clinical observations associated bacterial taxa at genus (A) and species (B) level identified by LEfSe (LDA score > 4).**


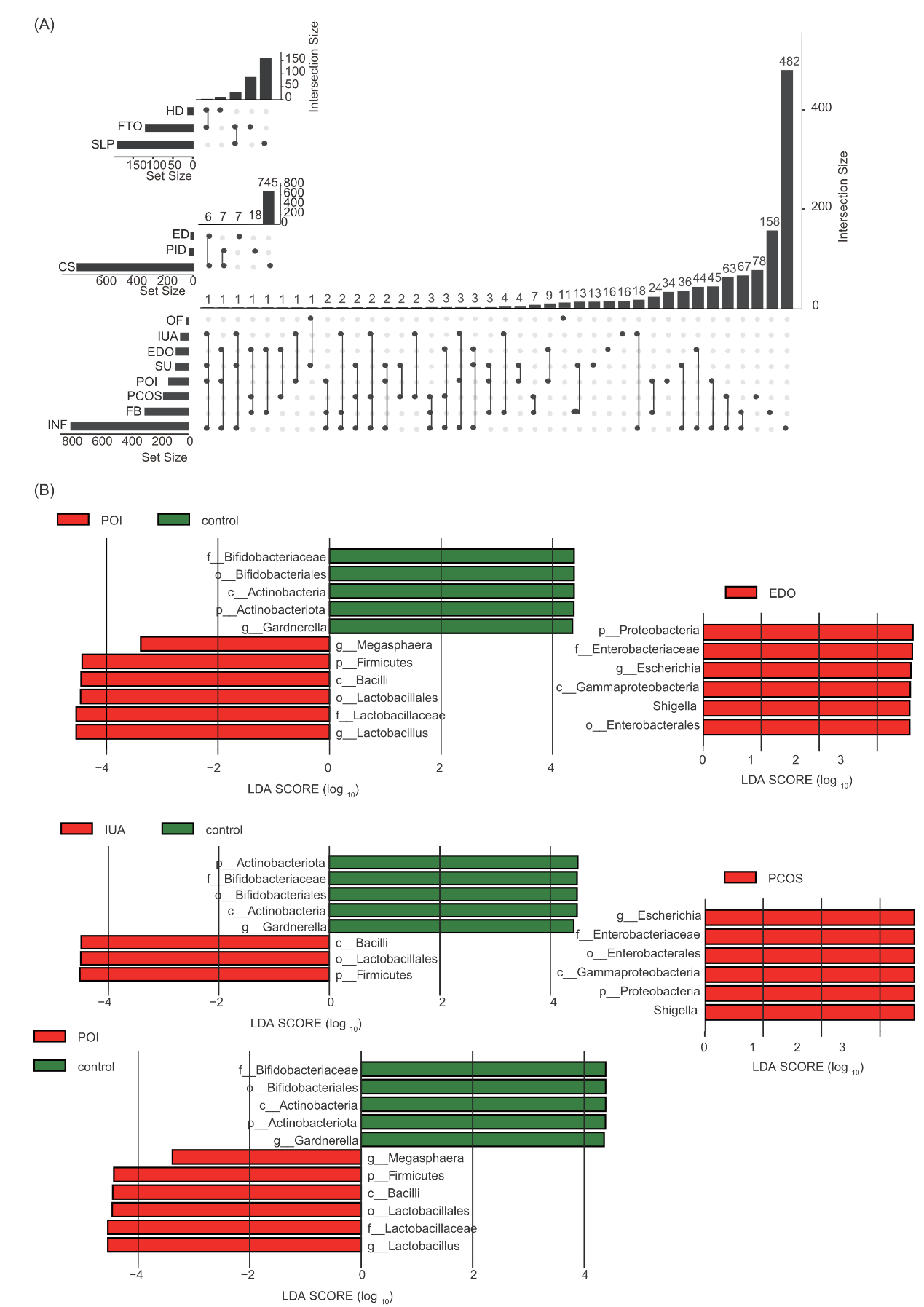


**Figure S5 Characteristics of each type of infertility.** (A) UpSet plot of the intersection of the women with each type of infertility. (B) Barplot showing infertility type associated bacterial taxa identified by LEfSe (LDA score > 4).


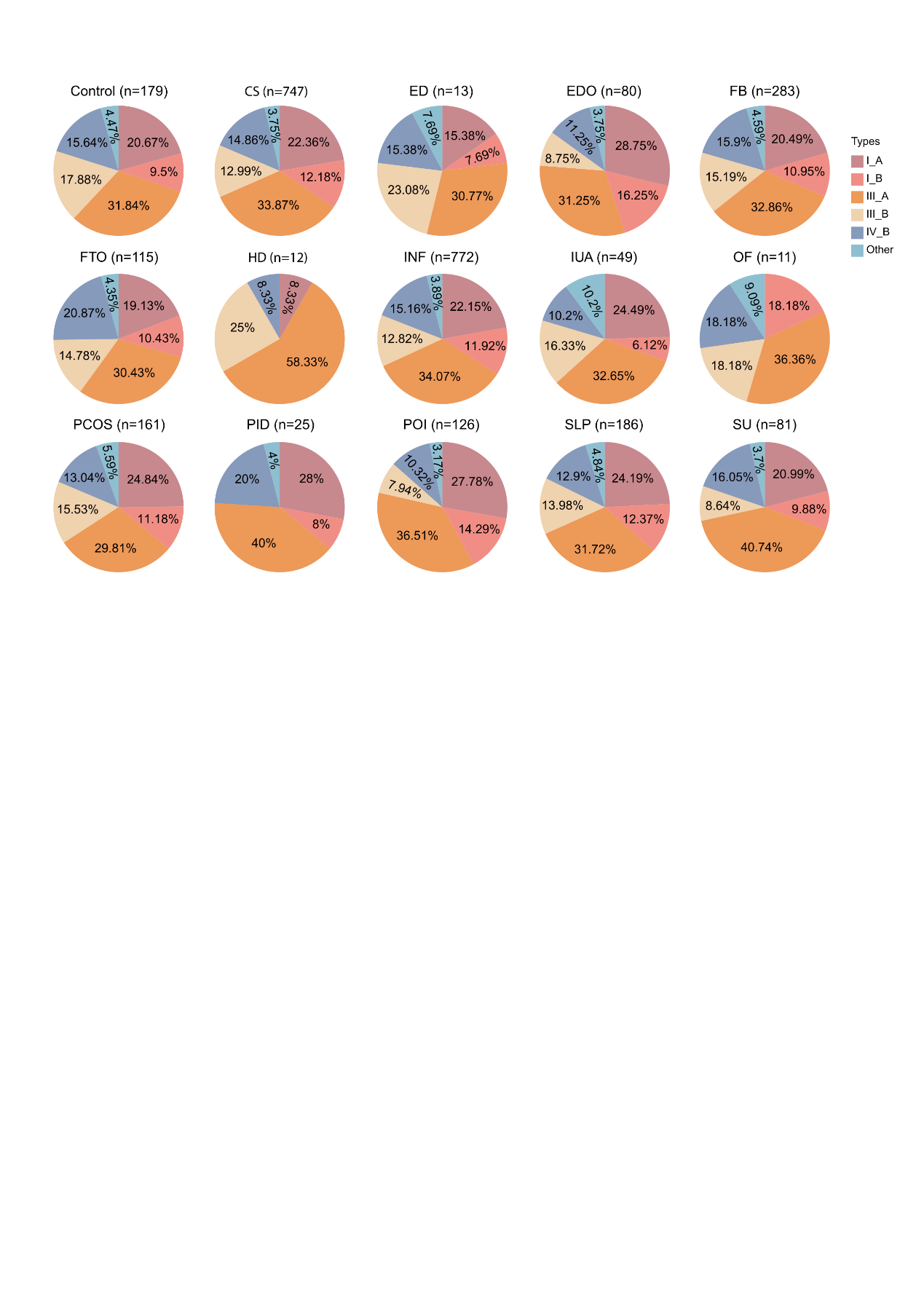


**Figure S6 Representation of vaginal bacterial community types (I-A, I-B, III-A, III-B, IV-B) within each group of women.** The number of women from each infertility group is in parentheses. Polycystic ovarian syndrome: PCOS; Premature ovarian insufﬁciency: POI; inflammatory: INF; intrauterine adhesion: IUA; endometriosis: EDO; scarred uterus: SU; ovulation failure: OF; fallopian problem: FB; chronic salpingitis: CS; endometritis: ED; pelvic inflammatory disease: PID; hydrosalpinx: HD; fallopian tube obstruction: FTO; salpingectomy: SLP. The data were analyzed by the Mann-Whitney U test.


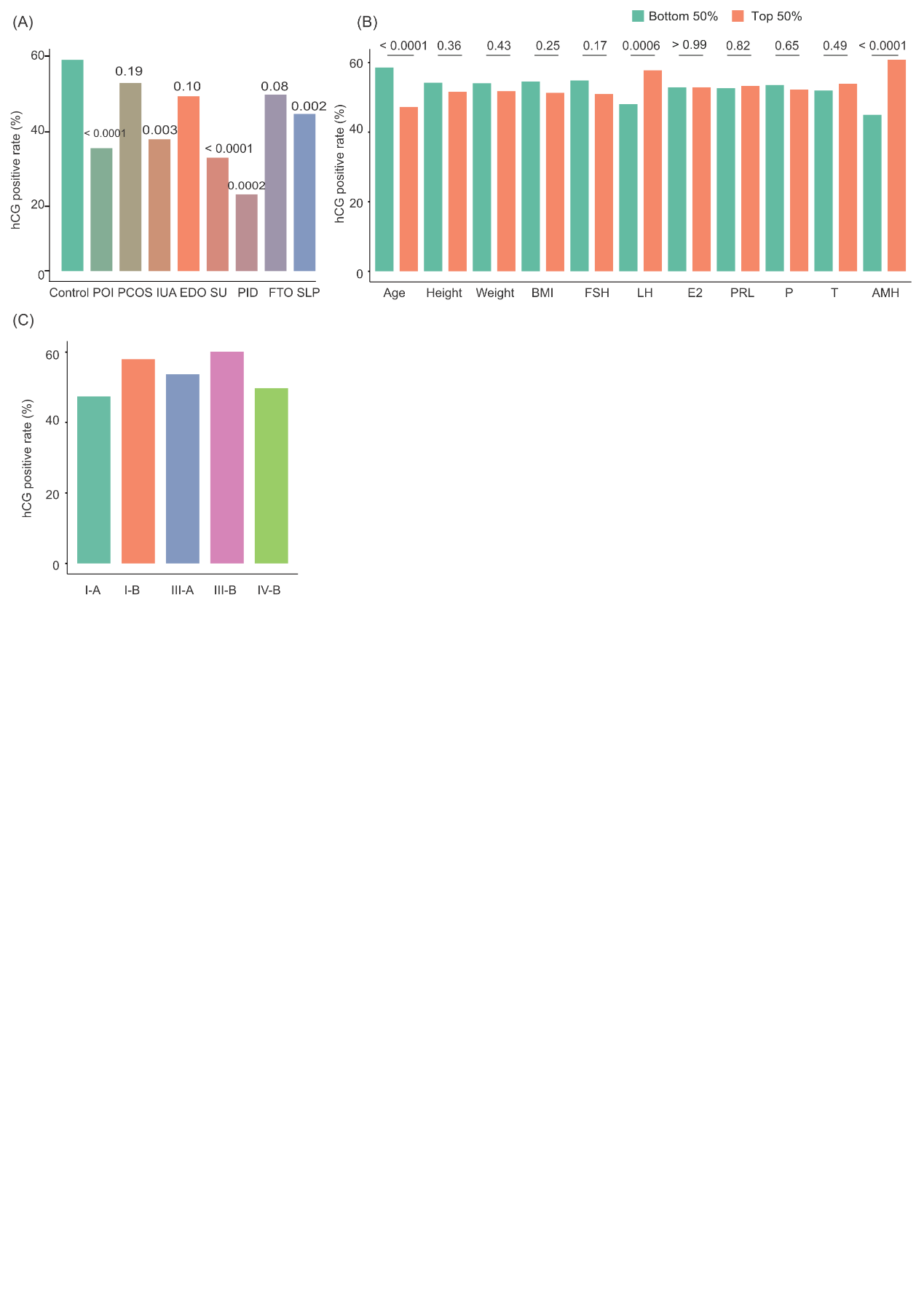


**Figure S7 Association of vaginal microbiota composition with IVF outcomes.** (A) Analysis of women who became hCG positive in each infertility group compared with control, some women were diagnosed with more than one symptom would be used to compute multiple times. (B) Analysis of hCG positive rate in top and bottom 50% sample for each measurement. Chi-square test was used to compare IVF outcomes between each group and control. (C) The distribution of hCG positive results for each CST.


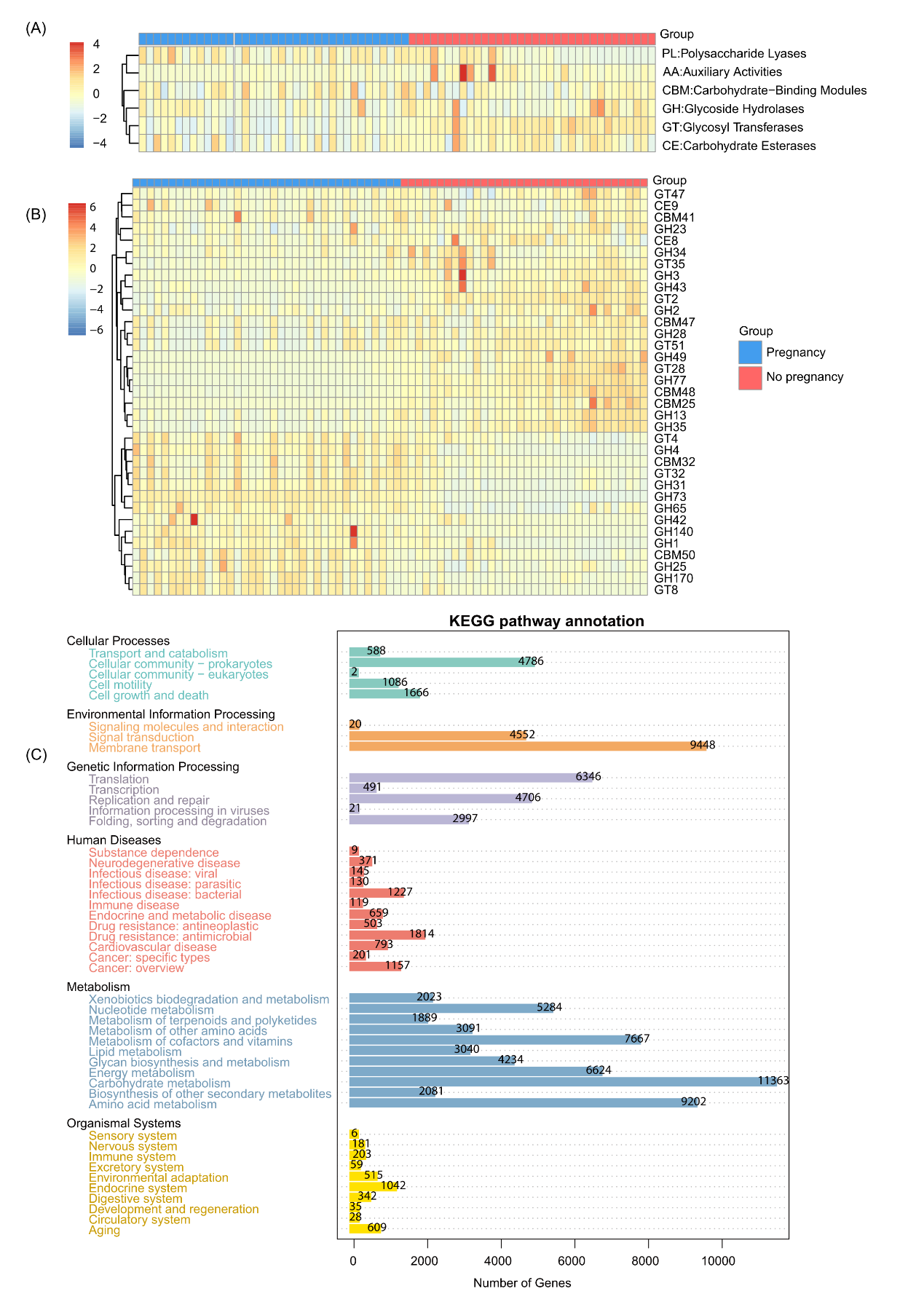


**Figure S8** **Function difference of vaginal metagenome between pregnant and non-pregnant women.** The relative abundances of functionally and phylogenetically annotated orthology classiﬁed based on the CAZy (A), eggNOG (B), and KEGG (C) database.
